# Therapeutic Potential of Targeting the JAK/STAT Pathway in Psoriasis: Focus on TYK2 Inhibition

**DOI:** 10.3390/jcm13113091

**Published:** 2024-05-24

**Authors:** Martina Dragotto, Martina D’Onghia, Emanuele Trovato, Linda Tognetti, Pietro Rubegni, Laura Calabrese

**Affiliations:** 1 Dermatology Unit, Department of Medical, Surgical and Neurological Sciences, University of Siena, 53100 Siena, Italyemanuele.trovato@ao-siena.toscana.it (E.T.); pietro.rubegni@unisi.it (P.R.); 2 Institute of Dermatology, Catholic University of the Sacred Heart, 00168 Rome, Italy

**Keywords:** psoriasis, JAK/STAT pathway, JAK inhibitors, TYK2 inhibitors

## Abstract

Psoriasis is an inflammatory skin disease with a chronic relapsing course and an often-detrimental impact on patients’ quality of life. Thanks to incredible advances in research over the past few decades, the therapeutic armamentarium of psoriasis is now reasonably broad and structured, with several therapeutic agents that have demonstrated successful long-term control of this condition. However, there are still unfulfilled gaps resulting from the inherent limitations of existing therapies, which have paved the way for the identification of new therapeutic strategies or the improvement of existing ones. A great deal of attention has recently been paid to the JAK/STAT pathway, playing a crucial role in chronic inflammatory skin diseases, including psoriasis. Indeed, in a disease with such a complex pathogenesis, the possibility to antagonize multiple molecular pathways via JAK/STAT inhibition offers an undeniable therapeutic advantage. However, data from clinical trials evaluating the use of oral JAK inhibitors in immune-mediated disorders, such as RA, have arisen safety concerns, suggesting a potentially increased risk of class-specific AEs such as infections, venous thromboembolism, and malignancies. New molecules are currently under investigation for the treatment of psoriasis, such as deucravacitinib, an oral selective inhibitor that binds to the regulatory domain of TYK2, brepocitinib (PF-06700841) and PF-06826647 that bind to the active site in the catalytic domain. Due to the selective TYK2 blockade allowing the inhibition of key cytokine-mediated signals, such as those induced by IL-12 and IL-23, anti-TYK2 agents appear to be very promising as the safety profile seems to be superior compared with pan-JAK inhibitors. The aim of our review is to thoroughly explore the rationale behind the usage of JAK inhibitors in PsO, their efficacy and safety profiles, with a special focus on oral TYK2 inhibitors, as well as to provide a forward-looking update on novel therapeutic strategies targeting the TYK2 pathway in psoriasis.

## 1. Introduction

Psoriasis (PsO) is a chronic immune-mediated skin disease whose pathogenesis is only partially elucidated and based on a complex interplay between environmental and genetic factors. PsO has a significant physical, emotional, and psychosocial impact on affected patients [1]. Its prevalence widely varies among populations, with approximately 3% of individuals affected in the United States [2], while higher rates are observed in regions such as the Faroe Islands [3]. Conversely, certain ethnicities, like the Japanese and indigenous groups such as Aboriginal Australians and South American Indians, exhibit lower prevalence rates or even a complete absence of PsO [4].

PsO can manifest at any age, and its prevalence steadily increases over the course of life [5,6]. The disease can present with several clinical variants, including plaque, guttate, inverse, erythrodermic, and pustular PsO [7]. Each variant typically exhibits distinct clinical features and usually predominates in each patient; however, they may sometimes coexist within an individual simultaneously [8]. Key clinical characteristics of PsO include erythema, thickening, and scaling [9]. Plaque PsO or PsO vulgaris is the most common type, and it is characterized by well-delineated scaly plaques that are red or salmon-pink, and may vary in size and thickness [4]. These plaques are generally symmetrically distributed and mostly located on the extensor surfaces of elbows and knees, lumbosacral region, umbilicus, and scalp [10]. Nail and oral mucosa involvement, including the tongue, may also occur, exhibiting distinctive patterns such as geographic tongue [11]. Furthermore, approximately 30% of patients with PsO may develop concurrent psoriatic arthritis (PsA), which can manifest as oligoarthritis, spondylitis, enthesitis, or dactylitis [12].

In addition, metabolic syndrome (MetS), hypertension, dyslipidemia, fibromyalgia, obesity, and atherosclerosis have presented a higher prevalence in the last few years in psoriatic patients [13,14].

PsO can manifest with different degrees of severity, typically assessed in daily clinical practice using specific scores like the PASI (Psoriasis Area and Severity Index) [15] or body surface area (BSA)

Thanks to incredible advances in research over the past few decades, the therapeutic armamentarium of PsO is now reasonably broad and structured, featuring numerous therapeutic agents that have proven effective for long-term management of the condition. In this context, the choice of the appropriate treatment option depends on various factors, including disease severity, comorbid conditions, prior antipsoriatic therapies, and the presence of concomitant PsA, with the aim of customizing the treatment for each patient.

Mild cases of PsO, usually affecting less than 3–5% of the BSA, can often be treated with topical therapies such as steroids, vitamin D analogues, calcineurin inhibitors, keratolytics, and phototherapy [16]. In contrast, systemic treatments are generally required for moderate (5–10% BSA) and severe (>10% BSA) PsO, as well as for cases involving the so called “sensitive and visible” areas like the face, palms and/or soles, and genital regions [17].

Systemic treatments for PsO include traditional immunosuppressive drugs like cyclosporine and methotrexate, retinoids, fumaric acid esters, and orally administered small molecules including apremilast. Recent advances in understanding the pathophysiology of PsO have identified new therapeutic targets, leading to the development of highly selective and efficacious biotechnological agents, such as anti-TNF, anti-IL-12/23, and anti-IL-17 agents, administered either subcutaneously or intravenously, which have revolutionized the therapeutic approach to moderate-to-severe PsO [18,19].

Despite the availability of numerous treatment options for plaque PsO, response rates can differ significantly, and a subset of patients remains resistant, intolerant, or contraindicated to various therapies. This highlights a continuing unmet need within the therapeutic paradigm.

Recently, a great deal of attention has been paid to targeting the Janus kinase (JAK)/signal transducer and activator of transcription (STAT) pathway, a paradigm of receptor-mediated signal transduction, that plays crucial role in chronic inflammatory skin diseases, including PsO and atopic dermatitis (AD) [18,20,21,22]. Indeed, in a disease with such a complex pathogenesis characterized by the activation of more than one immune pathway, an alternative therapeutic strategy employing agents with broader activity, such as JAK inhibitors (JAKis), may be promising to provide therapeutic benefits to a wider range of patients and could offer an undeniable therapeutic advantage.

The aim of our review is to thoroughly explore the rationale behind the usage of JAKis in PsO, their efficacy and safety profiles, with a special focus on TYK2 inhibitors, as well as to provide a forward-looking update on novel therapeutic strategies targeting the TYK2 pathway in PsO.

## 2. Overview of Psoriasis Pathogenesis

The pathogenesis of PsO has become a highly active area of scientific investigation. Once considered a primary keratinization disorder, it is now understood that immune system dysregulation is the central element in PsO pathogenesis, involving a complex interaction between genetic and immunological factors [23].

The defining characteristic of PsO is persistent inflammation, which leads to excessive proliferation and abnormal differentiation of keratinocytes [24]. The inflammatory pathways involved in plaque PsO and other clinical variants are numerous; they exhibit overlapping features but also distinct differences, contributing to significant variations in phenotypes and treatment responses [23].

The inflammatory process in PsO involves both innate and adaptive immunity, working in a complex feedback loop aimed at amplifying the inflammatory cascade. This results in characteristic histopathological changes, including keratinocyte proliferation with parakeratosis, angiogenesis, neutrophil infiltration, and the enrichment of Th1/Th17 cells [25,26].

The recognition of the IL-23/IL-17 axis as the primary pathogenic pathway in PsO has significantly advanced our understanding of the disease and revolutionized its treatment approach [27]. The initial responders in the immune system are the cells of innate immunity, such as macrophages and dendritic cells. These cells subsequently release several mediators, including TNF-α, IL-12, IL-23, and type I interferon (IFN). Specifically, IL-12 and IL-23 are primarily synthesized by myeloid dendritic cells (mDC) [28], promoting the differentiation of Th1 and Th17 subpopulations, respectively. Conversely, type I IFNs are produced by plasmacytoid dendritic cells (PDC) [29]. The activation of PDC initiates the inflammatory cascade in PsO through the secretion of type I IFN (IFN-α and IFN-β). Type I IFN signaling, particularly IFN-α, induces mDC maturation, serving as an upstream cytokine in the IL-23/IL-17 axis [30].

Both IL-23 and IL-12 play pivotal roles in PsO pathogenesis [14]. IL-23 promotes a persistent inflammatory environment by activating Th17 cells, which release proinflammatory cytokines like IL-17A, IL-17F, and IL-22. Conversely, IL-12 stimulates the production of various cytokines, including GM-CSF, TNF-alpha, and IFN-γ, contributing to Th1 cell development [31].

IL-23 and IL-12 exploit the JAK/STAT signal transduction pathway to exert their biological effects.

In detail, both IL-12 and IL-23 attach to their respective receptor complexes, consisting of a common chain (IL-12Rβ1) and a secondary chain known as IL-12Rβ2 and IL23R, respectively. Upon attaching to their receptors, both cytokines initiate the activation the same JAK/STAT signaling molecules (JAK2, TYK2, STAT1, STAT3, STAT4, and STAT5) [32]. Notably, STAT3 induces the expression of RORγ, a transcription factor which is in turn involved in enhancing IL-17A, IL-17F, IL-22, and IL-23R gene expression [33,34].

Therefore, the JAK/STAT pathway, regulating a multitude of cellular processes, emerges as a pivotal convergence point for cytokine signaling implicated in PsO.

## 3. Role of JAK/STAT and TYK2 Signaling Pathways in Psoriasis

The JAK/STAT signaling pathway, which is evolutionarily conserved, mediates cellular responses to a wide array of cytokines and growth factors [35]. These responses encompass various fundamental cellular processes, including proliferation, differentiation, migration, apoptosis, and cell survival. The specific outcome of the JAK/STAT pathway activation is dependent on factors such as the nature of the signal, the tissue context, and the cellular environment [36]. The JAK/STAT signaling pathway progresses through several sequential steps. Initially, cytokine binding triggers the dimerization of receptors and the subsequent coupling of Janus kinases. Subsequently, tyrosine residues on receptors are phosphorylated, creating binding sites for STAT proteins containing the SH2 domain [37]. Once recruited, STAT proteins undergo phosphorylation and activation, resulting in the formation of functional dimers. Ultimately, these activated STAT dimers translocate into the cell nucleus, where they regulate gene expression by binding to specific DNA elements [38]. Each JAK subtype contains a specific tyrosine residue for ATP-dependent phosphorylation. Typically, the enzyme is inactive and unable to bind ATP. When a cytokine binds to its receptor on the cell surface, the receptor undergoes a conformational change that allows ATP to access its binding site in the enzyme’s catalytic domain, enabling it to exert its functions [39]. There are four types of JAK proteins (JAK1, JAK2, JAK3, and TYK2) and seven STAT proteins (STAT1, STAT2, STAT3, STAT4, STAT5a, STAT5b, and STAT6), with each STAT protein capable of binding to various JAK family members, leading to proinflammatory cellular immune responses [40]. JAK1 pairs with JAK2, JAK3, and TYK2, leading to downstream signal transduction initiated by cytokine receptors [41]. Heterodimers consisting of JAK2 and TYK2 have a specific function in transmitting signals mediated by type I IFN, IL-12, and IL-23 [42]. Specifically, TYK2 functions as a non-receptor tyrosine kinase involved in signaling various cytokines and interferons, regulating processes such as cell growth, migration, and both innate and adaptive immune responses [43].

In detail, TYK2, interacts with receptors for various cytokines including those of the IL-12 and IL-23 family, the type I IFN family, the IL-6 family, and the IL-10 family [44]. Upon binding of these cytokines and subsequent dimerization of their receptor subunits, TYK2 undergoes phosphorylation and activation, along with its partner JAKs, JAK1 and/or JAK2 [45]. To better understand the role of TYK2, a study on TYK2-/- mice was conducted. Specifically, TYK2-/- mice displayed reduced responses to IFN α/β and IL-12 and a selective deficiency in STAT3 activation in these pathways [46]. Further studies on TYK2-/- mice confirmed that TYK2 is necessary for IL-12 and IL-23 responses and plays a role in enhancing the sensitivity of type I interferon responses, although it is not essential for IL-6 or IL-10 [44]. Comprehensively, these findings revealed that TYK2 plays a crucial role in regulating immunity, impacting both innate and adaptive immune responses.

TYK2 can modulate cytokine receptor signaling through both its enzymatic activity and a kinase-independent scaffolding function [47]. For example, TYK2 has been shown to be crucial for the surface expression of the human type I IFN receptor, regardless of its enzymatic activity [48].

TYK2 plays a pivotal role in the pathophysiology of PsO by modulating downstream signaling of receptors, particularly those binding to IL-12, IL-23, type I-α IFN, and IFN-β [49]. These cytokines govern the functions of diverse Th cells, including Th1, Th17, and Th22 lymphocytes, all of which are integral to shaping the PsO phenotype. Indeed, experimental findings have demonstrated that mice lacking TYK2 fail to develop epidermal hyperplasia following IL-23 activation, unlike their wild-type counterparts expressing TYK2 [42]. The absence of TYK2 potentially diminishes the production of PsO-related cytokines, as well as antibacterial peptides [50]. The current understanding of the significant role played by TYK2 in PsO underscores its attractiveness as a target for the development of tailored inhibitors.

## 4. JAK Inhibitors in Psoriasis

Nowadays, an enhanced understanding of the cytokine signaling through the JAK-STAT pathway, has led to increased applicability of the therapeutic interventions with JAKis in multiple diseases, including dermatological conditions [51]. Despite blocking this molecular cascade have shown promising results in treating both PsO and PsA, the use of JAK1/2/3 inhibitors in PsO have been primarily halted due to concerns regarding their effectiveness and safety ratio [52].

Within oral selective JAK1 inhibitors, itacitinib, abrocitinib, solcitinib, and ivarmacitinib have been investigated for the treatment of moderate-to-severe PsO in several randomized clinical trials (RCT).

The JAK1 selective inhibitor itacitinib (INCB039110) underwent a 12-week phase 2 randomized controlled trial (RCT), on 50 moderate-to-severe psoriatic patients [53]. At week 4, itacitinib at a dose of 200 mg two times a day (BID) or 600 mg once a day (QD), has demonstrated a statistically significant higher efficacy in terms of percentage of patients achieving the Physician Global Assessment (PGA) 0/1 score and PASI 75 response rates compared to placebo [53]. In this trial, itacitinib has shown a positive safety profile, with the most frequently reported adverse event (AE) represented by nasopharyngitis (18.4%) [53]. However, no phase III clinical trials are currently investigating itacitinib in PsO.

The efficacy and safety of abrocitinib (PF-04965842) and solcitinib (GSK2586184) have been investigated in two separate 12-week phase 2 RCTs, showing promising outcomes in patients with moderate-to-severe PsO [54,55]. At week 4, a greater number of patients achieved the PASI 75 response in the abrocitinib 200 mg BID group (60%), in comparison to groups receiving 200 mg QD (17%), 400 mg QD (50%), and placebo (17%) [54]. As for solcitinib, 57% of patients in the 400 mg QD group achieved a PASI 75 response, with a noticeable improvement in itching and quality of life across all doses (100 mg, 200 mg, and 400 mg), compared to placebo [55]. Statistically significant differences (*p* < 0.001) were observed for 400 mg solcitinib BID compared with placebo at weeks 4, 8, and 12 [55]. However, both JAKis are no longer being studied for PsO, since abrocitinib found broader application in AD, while the prosecution of studies on solcitinib was halted due to AEs. Specifically, across all treatment groups, headache, nasopharyngitis, nausea, diarrhea, fatigue, and upper abdominal pain were the most common AEs [55]. In addition, five serious AEs related to study treatments occurred, including ureteral calculus and thrombocytopenia [55]. Finally, ivarmacitinib (SHR0302) is currently under investigation in phase 3 study on 444 PsA patients, primarily aimed at assessing effectiveness and safety of this drug (NCT04957550). Results of this trial are not yet available.

Filgotinib, a selective JAK1 inhibitor with minimal JAK2 activity, which is currently approved in European Union and Japan, was primarily investigated in a phase 2 RCT for PsA [56]. In this trial conducted on 131 patients, filgotinib 200 mg demonstrated a significant efficacy in achieving American College of Rheumatology (ACR) 20, ACR50, and ACR70 responses, along with a cutaneous improvement measured as PASI75 at week 16, compared to placebo (80% versus 33%, respectively), with a treatment difference of 47% (95% CI 30·2–59·6, *p* < 0.0001) [56]. Additionally, filgotinib displayed a rapid onset of action and only mild-to-moderate AEs, such as nasopharyngitis and headache, with no statistically significant difference compared to the placebo group (57% vs. 59%) [56].

The effectiveness and safety of baricitinib, an oral small molecule targeting both JAK1 and JAK2, in PsO were investigated in a phase 2b RCT [57]. By week 12, greater mean changes from baseline in their PASI were reported in patients treated with 2 mg, 4 mg, 8 mg, and 10 mg daily, in comparison to placebo (*p* < 0.005). Furthermore, all baricitinib treated groups, except for the 2 mg dose, had higher rates of PASI 50 compared to placebo [57]. Despite being well tolerated over 24 weeks, dose-dependent alterations in laboratory values were observed. Currently, baricitinib is exclusively FDA- and EMA-approved for RA, AD, and alopecia areata, with no ongoing clinical studies assessing its use in PsO or PsA.

Ruxolitinib is another JAK1 and JAK2 inhibitor, currently approved by FDA and EMA in its oral formulation for the treatment of myelofibrosis and polycythemia vera. However, its use in PsO has been limited to topical cream formulations [58]. Three clinical trials involving a total of 253 participants (NCT00617994, NCT00820950, and NCT00778700) have tried to assess its efficacy and safety in PsO, showing a statistically significant improvement in total lesion score compared to vehicle [58]. Moreover, ruxolitinib was found to be non-inferior compared to calcipotriene and betamethasone dipropionate [58]. However, it is not approved for treating psoriatic disease and there are no ongoing studies for PsO treatment.

Peficitinib (ASP015K), a new oral inhibitor primarily targeting JAK3 over JAK1 and JAK 2, underwent a 6-week phase 2a study involving 124 patients with moderate-to-severe plaque PsO [59]. Considering different dosages, including 10 mg BID, 25 mg BID, 60 mg BID, 100 mg BID, and 50 mg QD, significant improvements in PASI scores were observed in comparison to placebo (*p* < 0.001 for all treatment groups). Similarly, improvements in PGA 0/1 and body surface area (BSA) scores were noted compared to placebo (all *p* < 0.001) [59]. In terms of safety, peficitinib was generally well tolerated in this trial, with no serious AEs reported [59]. However, currently, there are no ongoing clinical trials focused on psoriatic disease.

Tofacitinib, a JAK1/3 inhibitor, was FDA-approved in December 2017 for the treatment of patients with moderate-to-severe PsA and an inadequate response to conventional synthetic DMARDs (csDMARDs). Two double-blind placebo-controlled phase 3 RCTs (the Oral Psoriatic Arthritis triaL [OPAL] and the Broaden and Oral Psoriatic Arthritis triaL [OPAL Beyond]) [60,61] and a long-term extension analysis of up to 48 months (OPAL Balance) [62] have shown statistically significant improvements in American College of Rheumatology 20 (ACR 20) response at 3 months and over time (48 months) in patients treated with tofacitinib 5 mg and 10 mg twice daily. Although tofacitinib was not indicated for PsO treatment due to safety concerns, its clinical efficacy has been investigated in several phase I, II, and III clinical trials, showing higher PASI75 responses compared to placebo in moderate to severe plaque PsO [62,63,64,65,66]. Also, a topical formulation of tofacitinib has been tested in chronic plaque PsO in three distinct RCTs (NCT01831466, NCT00678561, and NCT01246583) [58].

Finally, the efficacy and safety of upadacitinib, an FDA-approved oral selective JAK1 inhibitor for PsA, have been assessed in two main phase 3 trials (SELECT-PsA 1 and SELECT-PsA 2) [67,68]. Those trials have demonstrated efficacy of upadacitinib in skin outcomes, including PASI75/90/100 and Static Investigator Global Assessment of Psoriasis of 0 or 1 (sIGA 0/1) response rates, suggesting promising results as a future possible treatment for PsO [67,68]. A list of JAKis that have been investigated in psoriasis is provided in Table 1.

## 5. TYK2 Inhibitors in Psoriasis: A New Promising Class of Therapeutic Agents

As previously mentioned, the TYK2 signaling pathway plays an essential role in psoriatic disease, since its inhibition breaks the link between IL-23 and IL-17 axis, which is crucial in PsO pathogenesis. For the first time in 2010, TYK2 was identified as a PsO susceptibly gene in a genome-wide association study, thus further supporting its importance as potential therapeutic target for PsO treatment [29]. Currently, three oral inhibitors targeting TYK2 are approved or in clinical development: deucravacitinib (FDA and PDMA approved), ropsacitinib, and brepocitinib (Table 2).

**Figure 1:**
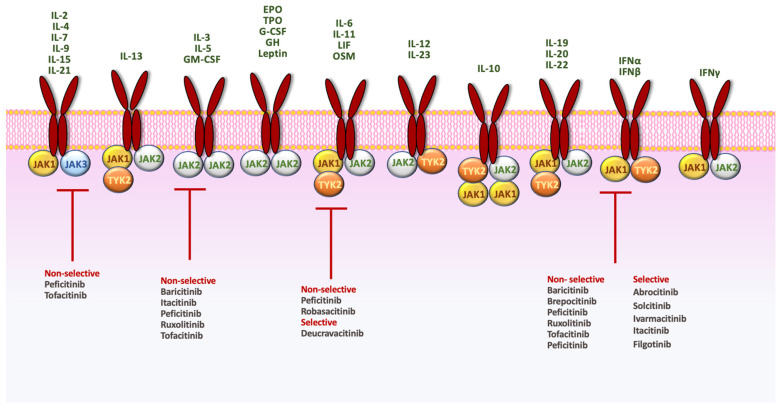
Schematic representation of mechanism of action and selectivity of JAK and TYK2 inhibitors. INF, interferon; IL, interleukin; OSM, oncostatin M; LIF, leukemia inhibitory factor; GM-CSF, granulocyte-macrophage colony-stimulating factor; C-CSF, granulocyte colony-stimulating factor; EPO, erythropoietin; TPO, thrombopoietin; GH, growth hormone. EPO: erythropoietin; G-CSF: granulocyte colony-stimulating factor; GH: growth hormone; GM-CSF: granulocite-macrophage colony stimulating factor; IFN: interferon; LIF: leukemia inhibitory factor; OSM: oncostatin M; TPO: thrombopoietin.

Figure 1 shows a schematic representation of the mechanisms of action and selectivity of the JAKis and TYK2 inhibitors investigated in moderate-to-severe PsO.

### 5.1. Deucravacitinib

Deucravacitinib (BMS-986165) is an orally, once-daily administered selective TYK2 inhibitor, approved in September 2022 at a dose of 6 mg QD by US-FDA for adult patients with moderate-to-severe plaque PsO, who are candidates for systemic therapy or phototherapy [69]. Deucravacitinib binds allosterically to the regulatory pseudo kinase domain of TYK2, unlike ropsacitinib, brepocitinib, and JAKis, which target the ATP-binding active site in the catalytic domain [70,71,72]. Thereby, deucravacitinib blocks the receptor-mediated conformational change of the catalytic domain required for ATP binding, effectively inhibiting TYK2 and preventing the downstream signaling of IL-23, IL-12, or type 1 interferons, which are primarily implicated in PsO pathogenesis [71,72].

Firstly, the effectiveness of deucravacitinib has been demonstrated in a phase 2 RCT (NCT02931838) involving patients with moderate-to-severe PsO. In this trial, a significantly higher proportion of patients treated with deucravacitinib 3 mg BID (69%), 6 mg BID (67%), and 12 mg QD (75%) therapy achieved PASI 75 at week 12, compared to placebo (7%; *p* < 0.001) [73].

Recently, two large phase 3 trials, POETYK PSO-1 (NCT03624127) and POETYK PSO-2 (NCT03611751), assessed the efficacy and safety of deucravacitinib, in comparison to apremilast and placebo, in adult patients with plaque PsO [74,75]. In total, those trials involved 1686 patients with moderate-to-severe PsO (666 in PSO-1 and 1020 in PSO-2, respectively) and ran over a period of 52 weeks. In both trials, participants were randomly assigned to receive either deucravacitinib 6 mg QD, apremilast 30 mg BID, or placebo (in a ratio of 2:1:1). At week 16, patients initially receiving placebo switched to deucravacitinib, while those receiving apremilast and experiencing relapse (measured as <PASI50 in PSO-1 and <PASI75 in PSO-2), switched to deucravacitinib at week 24.

At week 16, in both trials, the proportion of patients achieving PASI75 was significantly higher with deucravacitinib (58.7% in PSO-1 and 53.6% in PSO-2, respectively) compared to placebo (12.7% PSO1 and 9.4% PSO-2) or apremilast (35.1% PSO-1 and 40.2% PSO-2) (all *p* < 0.001). Similarly, PGA score 0/1 was obtained in 53.6% and 50.3% of patients treated with deucravacitinib, in contrast to 32.1% and 34.3% in the apremilast group and 7.2% and 8.6% of patients receiving placebo (all *p* = 0.0001) [74,75].

Interestingly, the improvement in PASI75 and sPGA 0/1 persisted up to week 24 in both studies, showing a greater effectiveness of deucravacitinib compared to apremilast. Specifically, at week 24, 69% and 59.3% (PSO-1 and PSO-2, respectively) of patients achieved a PASI 75 with deucravacitinib, in contrast to 38.1% and 37.8% with apremilast. Additionally, 58.4% and 50.4% of patients obtained an sPGA 0/1 response with deucravacitinib, compared to 31% and 29.5% with apremilast [74,75].

In addition, from POETYK PSO-1, it emerged that the response was maintained at week 52 in participants who received continuous deucravacitinib: 65.1% and 52.7% for PASI 75 and sPGA 0/1, respectively. Conversely, patients who switched from placebo to deucravacitinib at week 16 demonstrated PASI 75 and sPGA 0/1 responses at week 52 (68.3% and 53.8%, respectively) that were comparable to patients who received continuous deucravacitinib treatment [74]. Overall, deucravacitinib exhibited a good tolerability, with nasopharyngitis, headache, diarrhea, nausea, and upper respiratory tract infection reported as the most common AEs [74,75].

Consistently, those results were confirmed in a phase 3b RCT, the POETYK long-term extension (LTE) (NCT04036435), where deucravacitinib maintained efficacy and safety with no different AEs events as previously observed over 2 years [76].

Recently, Imafuku et al. [77] published a subgroup analysis from the POETYK PSO-1 study [74] assessing the efficacy and safety of decucravacitinib in a cohort of 66 Japanese patients. These patients were randomly assigned to receive either deucravacitinib 6 mg once daily (*n* = 32), placebo (*n* = 17), or apremilast 30 mg twice daily (*n* = 17). Remarkably, the efficacy and safety of deucravacitinib was consistent with previous findings from the POETYK PSO-1 [74].

Based on the results of POETYK PSO-1 and PSO-2 trials, POETYK PSO-4, an open-label, single-arm, phase 3 clinical trial (NCT03924427), confirmed the efficacy and safety of deucravacitinib 6 mg QD in 74 adult Japanese patients with plaque (PP, *n* = 63), generalized pustular (GPP, *n* = 3), and erythrodermic (EP, *n* = 8) PsO [78]. At week 16, 76.2%, 66.7%, and 37.5% of patients with PP, GPP, and EP, respectively, achieved PASI 75, and 82.5%, 0.0%, and 50.0% achieved sPGA 0/1. Responses were maintained overall through week 52. AEs occurred in 74.6% of patients with PP, 100% of patients with GPP, and 87.5% of patients with EP. The most common AEs were nasopharyngitis and acne.

Another phase 3 RCT (POETYK-PSO-3) is ongoing to evaluate the efficacy and safety of deucravacitinib 6 mg QD compared to placebo in subjects with moderate-to-severe plaque PsO in China, Taiwan, and South Korea. Primary outcomes of the study are represented by (sPGA) 0/1 response and PASI 75 response. Results are not available so far (NCT04167462).

### 5.2. Brepocitinib

Brepocitinib (PF-06700841) is a potent oral inhibitor that targets TYK2 and JAK1. This selective inhibition leads to the modulation of multiple cytokine pathways involved in the pathogenesis of several diseases. Specifically, brepocitinib has been proposed for the treatment of systemic lupus erythematosus, Crohn’s disease, ulcerative colitis (UC), alopecia areata (AA), vitiligo, and hidradenitis suppurativa (HS) [79,80,81]. Also, topical administration of this small molecule had been developed for atopic dermatitis [79]. Currently, brepocitinib is under development for psoriatic disorders [82]. In a phase 1 trial, dosages up to 100 mg QD were found to be effective and well-tolerated for moderate-to-severe plaque PsO [83]. In addition, a phase 2 RCT involving 212 psoriatic patients, has shown a statistically significant decreases in PASI score with both breprocitinib 30 mg QD and 60 mg QD doses, compared to placebo at week 12 [84]. Consistently, in a phase 2b RCT including 218 PsA patients, brepocitinib 30 mg and 60 mg QD demonstrated superior efficacy in PASI75 and PASI90 at week 16, compared to placebo, with sustained responses over 52 weeks [85]. Interestingly, mild AEs were reported in 66% of cases through week 16, while no major AEs such as cardiovascular events, thromboembolic events, serious infections, or deaths occurred up until week 52 [85]. Finally, a phase 2b RCT demonstrated that topical brepocitinib was well tolerated but did not result in significant changes in PASI scores, when administered to mild-to-moderate PsO patients, compared with vehicle [86].

### 5.3. Ropsacitinb

Ropsacitinib (PF-06826647), an orthosteric competitive catalytic-site inhibitor that binds TYK2 and JAK2, was firstly investigated in a phase 1 trial, in which 40 patients with moderate-to-severe PsO were randomized to ropsacitinib (100 mg or 400 mg) or placebo QD for 28 days [87]. Overall, ropsacitinib showed good efficacy in reducing disease activity after 28 days in terms of PASI 75, target plaque severity score, and BSA [87]. In a phase 2b RCT on 178 moderate-to-severe plaque PsO patients, oral ropsacitinib was randomly assigned once daily (1:1:2:2:2) to 50 mg, 100 mg, 200 mg, 400 mg, or placebo (16 weeks). Participants who completed the investigational treatment period and were previously randomized to 200 mg or 400 mg continued these doses, while those randomized to other doses were assigned to 200 mg or 400 mg once daily for 24 weeks [88]. At week 16, a significantly greater proportion of participants (risk difference % [90% CI]) achieved PASI90 in the 200 mg (33.0 [18.0, 47.1], *p* = 0.0004) and 400 mg (46.5 [30.6, 60.6], *p* < 0.0001) groups versus placebo [88]. By week 40, significant improvements over placebo were observed for PASI50/75/90/100 and PGA 0/1 (200 and 400 mg; weeks 6–16; *p* < 0.05). Ropsacitinib demonstrated favorable tolerability throughout the 40-week period, with most treatment-emergent AEs being categorized as mild or moderate [88].

## 6. Conclusions

PsO is a chronic inflammatory skin condition whose pathogenesis is mainly driven by the IL23/1L-17 axis. Biological agents provided an excellent treatment option for patients with PsO so far, as they offer a highly selective approach with a superior safety profile compared with traditional systemic agents. Currently approved biologics target extracellular cytokines that are crucial in PsO pathogenesis, such as TNF-α (adalimumab, infliximab, certolizumab and etanercept), IL-23 (ustekinumab, guselkumab, tildrakizumab and risankizumab), and IL-17 (secukinumab, ixekizumab, bimekizumab and brodalumab). Their use has revolutionized the therapeutic landscape of PsO but is still hampered by certain limitations, including cost-related issues. Moreover, response rates may vary considerably and there is still a proportion of patients resistant, intolerant, or contraindicated to multiple therapies, representing a persistent unmet clinical need.

Recently, a great deal of attention has been paid to JAK/STAT inhibitors, since the possibility to antagonize multiple molecular pathways offers an undeniable therapeutic advantage in a complex disease such psoriasis. Additionally, the oral formulation of those drugs may bring some advantages, such as no risk of immunogenicity or injection-site reactions, but also easier transportation and storage. However, data from clinical trials assessing the administration of oral JAKis in immune-mediated disorders, such as RA, have arisen safety concerns. Indeed, these trials suggested a potential increase in the risk of class-specific AEs, including serious infections, venous thromboembolism, major adverse cardiovascular events, and malignancies. Nevertheless, in other conditions, such as AD, those risks were not entirely confirmed, thus highlighting how differences in safety profiles might also mirror differences in patient characteristics across several diseases [89]. TYK2 inhibitors, such as deucravactinib, brepocitinib (PF-06700841), and ropsacitinib (PF-06826647), are a promising new class of molecules that have been recently investigated for PsO, showing a rapid onset of clinical responses and early improvements in this class of patients. A higher selectivity for TYK2 over JAK 1, 2, and 3 allows for the inhibition of crucial cytokine-mediated signals, such as those induced by IL-12 and IL-23, thereby reducing the likelihood of off-target effects. Indeed, there were no alarming reports of herpes zoster, opportunistic infections, thromboembolic events in TYK2 inhibitors clinical trials, in contrast to those on JAK 1, 2, and 3 inhibitors, both in PsO and in other diseases [49,90]. Furthermore, no clinically significant abnormalities in laboratory parameters were observed with deucravacitinib treatment, suggesting that routine laboratory monitoring during deucravacitinib treatment may not be strictly necessary [49,90].

On this background, anti-TYK2 agents could theoretically claim a better safety profile compared with JAKis that interact with multiple molecular pathways. However, further research and surveillance are necessary to validate the properties of these drugs in order to improve the individualized approach for the management of this challenging condition as well as to better delineate their safety profile in comparison to JAKis. 

## Figures and Tables

**Table 1:** Emerging JAK inhibiting agents for moderate-to-severe psoriasis.

Drug, Trial	MoA	Clinical Trial No	Phase of Clinical Trials	Status	Approved for PsO
Itacitinib, (INCB039110)	JAK1 and JAK2 inhibition	NCT01634087	Phase II	Completed	None
Abrocitinib (PF-04965842)	JAK1 inhibition	NCT02201524	Phase II	Completed	None
Solcitinib (GSK2586184)	JAK1 inhibition	NCT01782664	Phase II	Completed	None
Baricitinib	JAK1 and JAK2 inhibition	NCT01490632	Phase II	Completed	None
Ruxolitinib	JAK1 and JAK2 inhibition	NCT00617994	Phase II	Completed	None
		NCT00820950	Phase II	Completed	
		NCT00778700	Phase II	Completed	
Tofacitinib	JAK 1 and JAK 3 inhibition	NCT02193815	Phase I	Completed	
		NCT01736696	Phase I	Completed	
		NCT00678561	Phase II	Completed	
		NCT01246583	Phase II	Completed	
		NCT01831466	Phase II	Completed	
		NCT01710046	Phase II	Completed	
		NCT00678210	Phase II	Completed	
		NCT01163253	Phase III	Terminated	
		NCT01815424	Phase III	Completed	
		NCT01276639	Phase III	Completed	
		NCT01309737	Phase III	Completed	
		NCT01519089	Phase III	Completed	
		NCT01186744	Phase III	Completed	
		NCT01241591	Phase III	Completed	
Peficitinib (ASP015K)	JAK1, JAK2 and JAK3 inhibition	NCT01096862	Phase II	Completed	None

Legend: MoA, mechanism of action; PsO, Psoriasis.

**Table 2:** Emerging TYK2 inhibiting agents for moderate-to-severe psoriasis.

Drug Name	MoA	Clinical Trial No.	Study	Status	Primary Endpoint(s)
Deucravacitinib (BMS-986165)	Selective TYK2 inhibitor	NCT02931838	Phase II, randomized, double-blind, placebo controlled	Completed	Proportion of participants achieving PASI75 by week 12; proportion of patients with AEs
		NCT03624127	Phase III, randomized, double-blind, placebo-controlled (POETYK PSO-1)	Completed	Proportion of participants receiving deucravacitinib with a sPGA score of 0 or 1 compared to placebo by week 16; proportion of participants achieving PASI75 by week 16
		NCT03611751	Phase III, randomized, double-blind, placebo-controlled (POETYK PSO-2)	Completed	Proportion of participants receiving deucravacitinib with a sPGA score of 0 or 1 compared to placebo by week 16; proportion of participants achieving PASI75 by week 16
		NCT04036435	Phase III, open-label, extension (POETYK long-term extension) (LTE)	Active, not recruiting	Incidence of AEs and serious AEs
		NCT03924427	Phase III, open-label, single-arm (POETYK PSO-4)	Completed	Proportion of participants receiving deucravacitinib with a sPGA score of 0 or 1 compared to placebo by week 16; proportion of participants achieving PASI75 by week 16
		NCT04167462	Phase III, randomized, double-blind, placebo-controlled (POETYK PSO-3)	Completed	Proportion of participants receiving deucravacitinib with a sPGA score of 0 or 1 compared to placebo by week 16; proportion of participants achieving PASI75 by week 16
Brepocitinib (PF-06700841)	Dual TYK2/JAK1 inhibitor	NCT02310750	Phase I, randomized, double blind, placebo-controlled, parallel group	Completed	Pharmacokinetics and pharmacodynamics outcomes
		NCT02969018	Phase IIa, randomized, double-blind, placebo-controlled	Completed	PASI score by week 12
		NCT03963401	Phase IIb, randomized, double-blind, placebo-controlled	Completed	Proportion of participants achieving an ACR 20 response by week 16
		NCT03850483	Phase IIb, randomized, double-blind, vehicle-controlled, parallel group	Completed	PASI score by week 12
Ropsacitinib (PF- 06826647)	Dual TYK2/JAK2 inhibitor	NCT03210961	Phase I, randomized, double-blind, placebo-controlled	Completed	Incidence of AEs and clinical laboratory abnormalities
		NCT03895372	Phase IIb, randomized, double-blind, placebo controlled, parallel-group	Completed	Proportion of participants achieving PASI 90 by week 16; incidence of AEs, clinically significant changes in vital signs, laboratory tests results and treatment-emergent electrocardiogram (ECG) findings by 40 weeks

Legend: ACR, American College of Rheumatology; AE, adverse event; MoA, mechanism of action; PsO, psoriasis; PASI, Psoriasis Area and Severity Index; sPGA, Static Physician’s Global Assessment.
